# Direct delivery of MRI contrast through skull vessel/marrow pathways into the brain guided by microCT

**DOI:** 10.7150/thno.117250

**Published:** 2025-06-09

**Authors:** Li Liu, Martin J. MacKinnon, Tatjana Atanasijevic, Stephen Dodd, Nadia Bouraoud, Danielle Donahue, Harikrishna Rallapalli, Alan P. Koretsky

**Affiliations:** 1 Section on Plasticity and Imaging of the Nervous System, Laboratory of Functional and Molecular Imaging, National Institute of Neurological Disorders and Stroke, National Institutes of Health, Bethesda, MD 20892, USA.; 2 Mouse Imaging Facility, National Institute of Neurological Disorders and Stroke, National Institutes of Health, Bethesda, MD 20892, USA.

**Keywords:** direct/transcranial brain drug delivery, skull vessel/marrow pathways, high resolution microCT, manganese-enhanced MRI, manganese ion (Mn^2+^)

## Abstract

**Rationale:** The brain remains a challenging organ for drug delivery. Earlier studies demonstrated that transcranial application of small molecular therapeutics and MRI contrast such as manganese ion (Mn^2+^) could serve as a new method for delivering molecules to the brain. In this earlier work using rats, manganese-enhanced MRI (MEMRI) demonstrated that Mn^2+^ passed most effectively through regions of the skull containing suture lines or dense vessel/marrow. In the present study, the delivery of Mn^2+^ to the brain using specific skull vessel/marrow pathways has been investigated.

**Methods:** *In-vivo* microCT scans of rat skull was conducted to study the intricate geometry of vessel/marrow pathways connecting the outer skull surface and meninges. Specific vessel/marrow paths were identified. MnCl_2_ (500 mM) solution was pipetted directly on the skull bone surface above the target path. After 2 hr, rats were subjected to MRI.

**Results:** High-resolution microCT images reveal that (a) there are "short paths" through the skull which have vessels on the outer surface of the skull, which directly pass through the vessel/marrow and then reach the meninges on the other side of the skull; (b) the skull above the cerebellum (interparietal bone) exhibits a significantly higher density of vessel/marrow compared to the frontal and parietal bone enabling testing whether direct application to skull enables transcranial movement and (c) thinning the skull in specific regions can lead to exposing vessel pathways from mid-skull to the meninges. Guided by microCT imaging, Mn^2+^ delivery to the brain could be accomplished as assayed with MEMRI through these different specific pathways. Two hours post pipetting MnCl_2_ solution onto the top of a short path through the intact skull, Mn^2+^ could be delivered readily to levels that produce detectable brain tissue enhancement by MEMRI. A T_1_ enhanced volume of 2.27 ± 1.47 mm^3^ was measured through the short path. Two hours post applying a MnCl_2_ solution to the intact skull above the cerebellum enabled MEMRI detection of a volume of enhanced brain tissue of 2.48 ± 2.66 mm^3^. Finally, in areas where surface short paths are absent but a path from mid skull to meninges is present, minimal thinning of the skull led to effective Mn^2+^ delivery, enabling MEMRI detection of volume of 4.68 ± 2.70 mm^3^.

**Conclusions:** MicroCT-guided transcranial delivery via vessel/marrow pathways may offer a less invasive and more localized method for administering imaging probes and therapeutics to the brain.

## Introduction

Previous studies showed that transcranial application can be a new brain delivery method for small molecular imaging agents and therapeutics, such as manganese ions (Mn^2+^), fluorescent molecules, dextrans, glutathione, and several purinergic receptor inhibitors 1, 2. The efficiency of transcranial delivery through the intact rat skull could be detected by MRI using skull application of MnCl_2_, to enable imaging small molecules penetration of the skull 1, 2. As detected by manganese-enhanced MRI (MEMRI) 3, Mn^2+^ was found to pass most efficiently through areas of the skull that contain suture lines such as bregma and lambda, compared with normal skull bone 2. It also passed through areas that had a high density of vessel and marrow in the skull.

There has been a renaissance in interest in the connections between skull and brain. The skull contains a complicated network of vessels and marrow. There has been work that demonstrates that immune cells can leave the skull marrow and enter directly into brain circulation 4-7. Furthermore, there has been interest in bacteria and tumor cells infiltrating the brain via vessel/marrow pathways through the skull 8, 9. Recently, it was reported that skull marrow and brain meninges are connected and regulated by vessels that bridge the skull and meninges. The perivascular space cerebrospinal fluid (CSF) can pass directly between skull marrow and the subarachnoid space via these skull-dura channels 6, 10, 11. These reports lend support to the previous work showing that small molecules and MRI contrast agents such as Mn^2+^ can pass through the skull, traverse the meninges and get into the brain and that MEMRI provides a good tool to study passage through skull vessel/marrow pathways 1, 2, although the mechanism whereby Mn^2+^ penetrates the meninges into brain is still unclear.

Recently, transcranial delivery methods are being tested to administer small-molecule psychiatric, neuroprotective, anti-cancer agents, and stroke therapies using preclinical mouse and rat models 12-14. In the delivery study using a mouse model, the concentration of small molecule drugs in the brain achieved through transcranial or “intraosseous” delivery was significantly higher than that obtained through intravenous administration — 115 times higher for risperidone and 294 times higher for paclitaxel. In these studies, skull bone was thinned to deliver drugs without regard to the vessel/marrow structure.

The aim of the present study was to determine if transcranial delivery through specific vessel/marrow pathways could be used at a variety of skull bone locations without regard to the overall density of vessel/marrow in the bone which limited earlier work 1, 2. This would enable MRI contrast and small therapeutics to be delivered to more specific locations in the brain. To identify specific vessel marrow pathways, high resolution micro computed tomography (microCT) was used to identify different useful geometries for delivering Mn^2+^ to the brain. MnCl_2_ was applied in an appropriate manner depending on the specific geometry used. MEMRI was used to monitor the delivery efficiency of Mn^2+^ in the brain tissue in terms of the amount of tissue that accumulated Mn^2+^ to get MEMRI contrast. Three different geometries were used. The first geometry was to take advantage of short, direct paths where a vessel enters the skull, enters the mid marrow/vessel region and a vessel continues directly through the skull and enters the meninges. Placing MnCl_2_ directly on top of the short path on the outer skull led to efficient development of MEMRI contrast. The second geometry was the skull above the cerebellum which contains a high density of vessel/marrow that connect to the meninges. Mn^2+^ can pass through the intact skull above the cerebellum simply by placing solution on the surface. Finally, and most invasively, microCT was used to find a path from mid skull over a vessel that passed directly to the meninges. CT-guided thinning of skull to about middle of the skull directly on top of the vessels that enters the meninges also led to increased MEMRI enhancement. In conclusion the specific geometry of vessel/marrow paths in the skull can be used to deliver Mn^2+^ through skull to brain tissues. The mechanism whereby Mn^2+^ penetrates the meninges is not clear, however, this work demonstrates that the specific vascular/marrow structure of the skull can be used as a brain delivery route.

## Materials and Methods

### Animal preparation

In this study, adult male Sprague-Dawley rats (age 7-10 weeks, body weights 200-300 g, n = 48 total), purchased from Harlan Laboratories, were used. There is no evidence that any of the results presented would be different in females so only males were used. All rats were handled in accordance with the Institute for Laboratory Animal Research guidelines and study procedures were approved by the Animal Care and Use Committee of the National Institute of Neurological Disorders and Stroke. A micro-CT scan (see below for details) was first obtained to identify the target vessel/marrow region. The rats were placed in an induction box and anesthetized with 3-5% isoflurane before being moved to a stereotaxic apparatus. After the rats were positioned in a stereotaxic apparatus, isoflurane was administered through a nose cone and adjusted between 1-3% as required, depending on the monitored respiratory rate of the animals. A single incision with a sterile scalpel was made through the skin of the skull at the target location. The skull bone was exposed by scraping away the periosteum. Sterile saline (0.9% NaCl) solutions of MnCl_2_ (500 mM, Sigma-Aldrich), were pipetted directly on the skull bone in 5 µL aliquots. Only saline was used in controls. The solutions were pipetted on the rat skull over a 2 hr period in 5 μL aliquots and replenished as needed. The total volume of applied MnCl_2_ solutions did not exceed 25 μL. After 2 hr, skin was sutured, and rats were subjected to MRI (see below for details). After MRI scan, rats were euthanized.

### MicroCT acquisition and analysis

*In-vivo* microCT scans of rats were conducted on a Bruker Skyscan 1176 micro-CT (Bruker microCT, Kontich, Belgium). Rats were anesthetized as described above. Scans were performed utilizing a 1 mm aluminum filter, with the X-ray source biased at 90 kV and 278 uA, and projections taken over 197 degrees at 1 of 2 scan settings. Most scans were performed using a binning factor of 1, with an 8.74 μm image pixel size (reconstructed into a final isotropic voxel size of 8.74 μm), obtaining projections every 0.3^o^, with 3 averages, each exposed at 1500 ms. Additional scans were performed using a binning factor of 2, with a 17.49 μm pixel size (reconstructed to an isotropic voxel size of 17.49 μm), obtaining projections ever 0.5^o^, with 3 averages, each exposed for 325 ms. Acquired data was reconstructed using NRecon (Bruker microCT, Kontich, Belguim), applying a nominal ring artifact reduction and beam hardening correction, as well as a dynamic contrast range, all optimized per scan to ideally distinguish the boundary between bone and soft tissue. Reconstructed images were rotated in DataViewer (Bruker microCT, Kontich, Belguim) along all 3 planes to mimic their orientation in the stereotaxic apparatus that would be used for Mn^2+^ delivery. Slices were reviewed to identify the approach for Mn^2+^ delivery and coordinates from the bregma or lambda to the desired location were recorded.

*Ex-vivo* high-resolution scans of rat skulls were conducted with a Skyscan1172 micro-CT (Bruker microCT, Kontich, Belgium). Scans were performed utilizing a 0.5 mm aluminum filter, with X-ray source biased at 65 kV and 153 uA. The image pixel size was 6.87 μm (reconstructed to an isotropic voxel size of 6.87 μm), obtaining projections ever 0.4^o^, with 3 averages, each exposed for 295 ms. Acquired data was reconstructed using NRecon as described above. The quantification of volume ratio of channel (total of skull surface to brain and brain to skull) to bone and marrow to bone was carried out by using ImageJ (https://imagej.net/ij/). Regions of interest (ROIs) were drawn manually.

*Ex-vivo* high-resolution scans of human skull was conducted with a Skyscan1176 micro-CT (Bruker microCT, Kontich, Belgium). Scans were performed utilizing a 0.1 mm copper filter, with X-ray source biased at 90 kV and 270 uA. The image pixel size was 36.13 μm (reconstructed to an isotropic voxel size of 36.13 μm), obtaining projections ever 0.7^o^, with 4 averages, each exposed for 23 ms. Acquired data was reconstructed using NRecon as described above.

### MRI acquisition

MRI experiments were performed on an 11.7 T animal MRI system (11.7 T/30 cm horizontal magnet, Magnex Scientific; MRI Electronics, Bruker Biospin) with a 12 cm integrated gradient shim system (Resonance Research Inc) using a 9 cm custom-built birdcage coil for signal transmission and a 2 cm custom-built surface coil placed on the rat head for signal reception. Rats were imaged 2 hr post MnCl_2_ application. Whole-brain T_1_-weighted spin echo pulse sequences were used for acquisitions. The following parameters were used: TR/TE = 500/7.6 ms, receiver bandwidth = 100 kHz, field of view (FOV) = 19.2 x 28.8 mm, matrix size = 192 x 288, number of slices = 30, slice thickness = 1mm, averages = 8. A subset of animals that received delivery of Mn^2+^ via the cerebellum and a thinned skull underwent partial-brain imaging. One animal in the cerebellum group was imaged with: TR/TE = 4000/8.9 ms, receiver bandwidth = 50 kHz, (FOV) = 30 x 30 mm, matrix size = 300 x 300, number of slices = 17, slice thickness = 0.6 mm, averages = 2. Two animals in the cerebellum group were imaged with: TR/TE = 800/6 ms, receiver bandwidth = 81.5 kHz, FOV = 30 x 30 mm, matrix size = 256 x 256, number of slices = 14, slice thickness = 0.6 mm, averages = 8. One animal in the thinned skull group was imaged with: TR/TE = 800/6.5 ms, receiver bandwidth = 75 kHz, field of view = 30 x 30 mm, matrix size = 300 x 300, number of slices = 14, slice thickness = 0.6 mm, RARE Factor = 2, averages = 2.

### MRI quantification

#### Preprocessing

MRI images were skull stripped with hand drawn brain masks using ITK-SNAP 15 before undergoing N4 bias field correction using the SimpleITK toolbox 16 to compensate for intensity non-uniformity. The Sigma *Ex-Vivo* Rat Brain template 17 was registered to each individual subject's image with Advanced Normalization Tools (ANT) 18. The template was aligned to the images with rigid-body, then affine, followed by non-linear SyN transformations using the ANTsRegistration function. To register the template to partial brain images, the template was cropped to match the anatomical coverage of the corresponding image using the tentorium cerebelli as a reference point. The Sigma *Ex-Vivo* Rat Brain Atlas was then warped into the individual subject space using the corresponding transformation parameters.

#### Analysis

To detect Mn^2+^-induced T_1_-shortening, we used a z-score threshold to detect high signal intensity voxels. Using the warped atlas, each subject's gray matter voxels were segmented. The z-score was calculated on a voxel-by-voxel basis by subtracting the mean signal intensity of the gray matter from each voxel, divided the standard deviation of the gray matter signal intensity. The calculation of z-score was restricted to gray matter voxels as signal intensity enhancements were not evident in the white matter. To identify high signal intensity voxels, a conservative z-score threshold of 2.58 was applied, corresponding to the top 0.5% of gray-matter signal intensities. Clustering was performed with the DBCAN algorithm to reduce the influence of noise and identify areas where Mn^2+^ penetrated. The DBSCAN algorithm assigns labels to a cluster based on a specified epsilon, which describes the maximum distance between points in a cluster and a minimum cluster size. Firstly, the distance of every suprathreshold voxel to its nearest neighbor was calculated. The optimal epsilon for the DBSCAN algorithm, was set as the 90^th^ percentile of the nearest neighbor distances. The minimum number of points in a cluster was set as the nearest integer value to epsilon. To avoid the influence of outliers, the median z-score within each subject's Mn^2+^-enhanced voxels was calculated. The 90^th^ percentile was calculated to capture the upper range of intensities, where Mn^2+^ delivery was most effective. The enhanced volume was calculated as the number of suprathreshold, Mn^2+^-enhanced, voxels multiplied by the individual voxel volume.

### Skull and femur bone histology

After rats were perfused with 5% formalin in PBS, their skull and femur bones were isolated. Bones were decalcified in EDTA (0.5 M, pH 8.0) for 10 days and cut into 5 µm section. H and E staining was conducted by Histoserv Inc. Light microscopic images were captured using a Nikon Eclipse Ti microscope.

### Statistical analysis

Statistical analysis was carried out with the one-way analysis of variance (ANOVA) with Tukey's multiple-comparisons test or unpaired t test. All statistical analyses were performed using GraphPad Prism 10.2.3.

## Results

**Figure 1:**
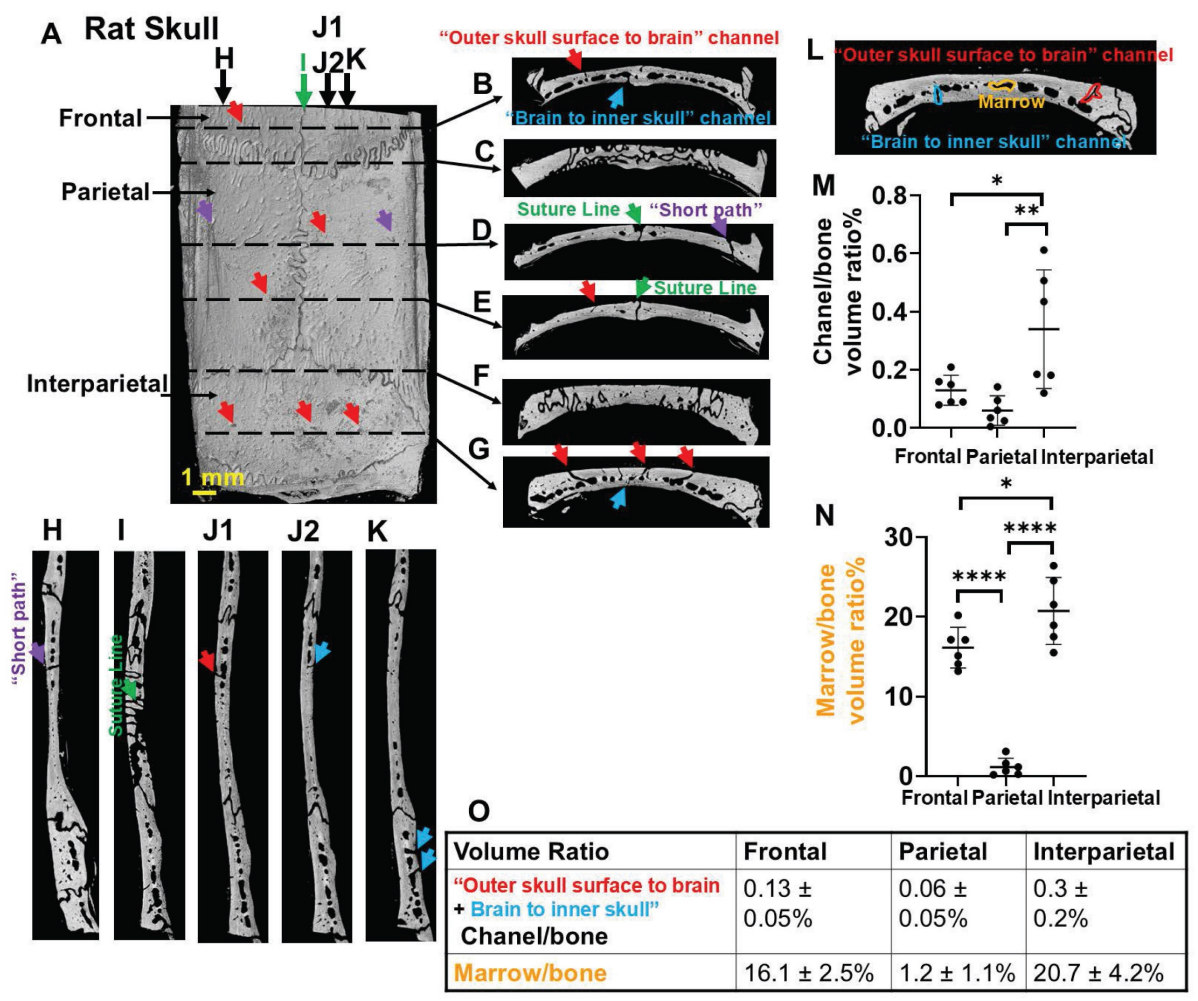
High-resolution microCT of a rat top skull showing an intricate geometry of vessel/marrow paths connecting the outer skull surface and meninges. (**A**) Outer skull surface. (**B-G**) Representative coronal views of the skull bone. (**H-K**) Representative sagittal views of the skull bone. (J1, J2) are two continuous images to show surface vessel/marrow reach the meninges. Red arrows, pores on the outer skull surface (**A**) indicating the “outer skull surface to brain” vessel/marrow pathways that get to the inner skull. Purple arrows, short paths that connect a vessel on the outer skull surface that connects to the mid skull vessel/marrow area with a vessel that connects to the meninges (**D**, **H**). Blue arrows, “brain to inner skull” vessel/marrow pathways. (**L-O**) Quantification of volume ratio of channel (total of skull surface to brain and brain to skull) to bone and marrow to bone. n = 6 rats. Results are expressed as mean ± SD. *, p < 0.05; **, p < 0.005; ****, p < 0.0001.

**Figure 2:**
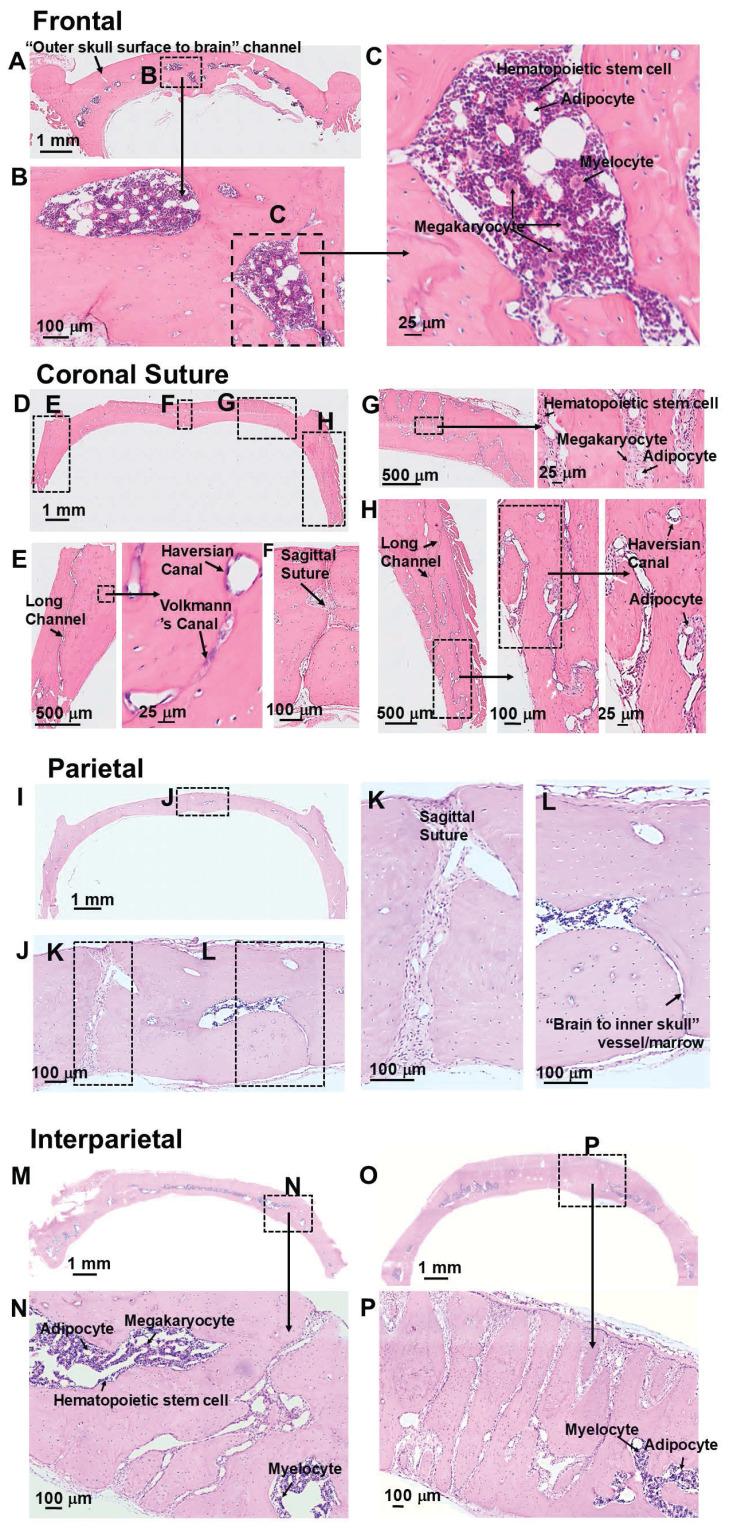
H and E staining of rat skull bone. (**A-C**) Frontal bone. (**D-H**) Coronal suture. (**I-L**) Parietal bone. (**M-P**) Interparietal bone.

**Animal number.** A total of 48 rats were used in this study, and Table S1 provides a summary of the number of animals used in each experiment. Briefly, 6 rats were used in Figure 1 for microCT study of skull and to quantify the volume ratios of channel to bone and marrow to bone. 6 rats were used in Figure 2 for H and E staining of rat skull bone. 9 rats were used in Figure 4 for Mn^2+^ delivery through “short paths” (n = 5 for Mn^2+^ delivery, n = 4 for saline control). 10 rats were used in Figure 5 for Mn^2+^ delivery above cerebellum (n = 6 for Mn^2+^ delivery, n = 4 for saline control). 12 rats were used in Figure 6 for Mn^2+^ delivery through minimum thinning of the skull (n = 6 for Mn^2+^ delivery, n = 6 for saline control). 1 rat was used in Supplementary Video 1 for reconstructed 3D microCT image of skull. 3 rats were used in Figure S1 for microCT of bottom skull. 1 rat was used in Figure S2 for H and E staining of femur bone marrow.

### An intricate geometry of vessel/marrow paths connects the outer skull surface and meninges

We used a rat model instead of a mouse model because, compared to the thinner mouse skull (0.1-0.3 mm), the rat skull (1-2 mm) more closely resembles the human skull (2-6 mm) and poses greater challenges for transcranial delivery. The skull is bright in microCT due to bone's high X-ray attenuation/radio-density (Figure 1). Vessels and marrow have poor X-ray attenuation, appearing dark and indistinguishable. At the suture lines there is a large space that traverses the top of the skull to the meninges that harbors large vessels (Figure 1D,E,I, green arrows). The normal skull bone also has an intricate pattern of many vessel/marrow paths connecting between the outer skull surface and meninges, as shown by high-resolution CT. Many pores can be observed from the outer skull surface (Figure 1A, red and purple arrows). If a path leaves the skull for the skin or leaves the skull for the meninges it was assumed to be a blood vessel. Thus, these pores indicate the vessels that go from skull to skin as well as connect deeper into vessel/marrow paths in the inner skull as shown in the coronal views and sagittal views (Figure 1B-L, red and purple arrows). Interestingly, there are "short pathways" in the skull that contain vessels on its outer surface, which travel directly through the vessel/marrow and connect to the meninges on the opposite side (Figure 1D,H, purple arrows). There are also many vessels from the inner skull that traverse into the meninges (Figure 1B-L, blue arrows). Figure 1 J1-J2 are two continuous images to show a surface vessel that goes fairly directly through mid-skull and then a vessel that gets directly into the meninges. The reconstructed 3D image to show the intricate geometry of vessel/marrow paths was shown in Supplementary Video 1 or Figshare (https://figshare.com/s/e506d8567974069184a9). The microCT cannot distinguish vessels from marrow so whether these direct pathways represent a single vessel or another vessel that comes from the mid region of skull that contains both marrow and vessels is not certain.

The skull above the cerebellum (interparietal bone) shows a significantly higher density of channels and vessel/marrow paths, compared with frontal and parietal bone (Figure 1L-O). The interparietal bone had 0.3 ± 0.2% volume fraction of vessels that leave the skull either to skin or to meninges. The volume fraction of mid skull marrow/vessels was 20.7 ± 4.2%. The frontal bone had a high density of mid skull marrow/vessels (16.1 ± 2.5%), but relatively lower density of vessels that leave the skull (0.13 ± 0.05%). The parietal bone had the lowest density of vessels that leave the skull (0.06 ± 0.05%) and mid skull marrow/vessels (1.2 ± 1.1%). From the microCT scan, it was also observed that the bottom skull contained a high density of vessel/marrow (Figure S1). While not demonstrated in this study it is likely that the bottom skull below the olfactory bulb and forebrain might provide other locations for transcranial delivery through the nose and mouth to the brain due to the high density of vessel/marrow.

H and E staining also showed the intricate geometries of skull marrow/vessel pathways (Figures 2 and S2). Consistent with the microCT results, frontal bone showed high density of marrow, but low density of vessels that leave the skulls (Figure 2A-C). Coronal suture showed high density of vessels that leave the skull (Figure 2D-H). Parietal bone showed low density of marrow cells and vessels that leave the skull. (Figure 2I-L). A representative view of a “brain to inner skull” vessel/marrow is shown in Figure 2L. Interparietal bone showed the highest density of marrow cells and vessels, with many channels connecting skull surface and brain surface (Figure 2M-P). More views of “outer skull surface to brain” vessel/marrow and “brain to inner skull” vessel/merrow at different locations of skull bones are shown in Figure S2 A-H. H and E staining of a femur bone is shown in Figure S2 I-K. In the bone marrow cells, hematopoietic stem cells, megakaryocytes, myelocytes, and adipocytes were observed. In the bone, Haversian canal and Volkmann's canal were observed.

**Figure 3:**
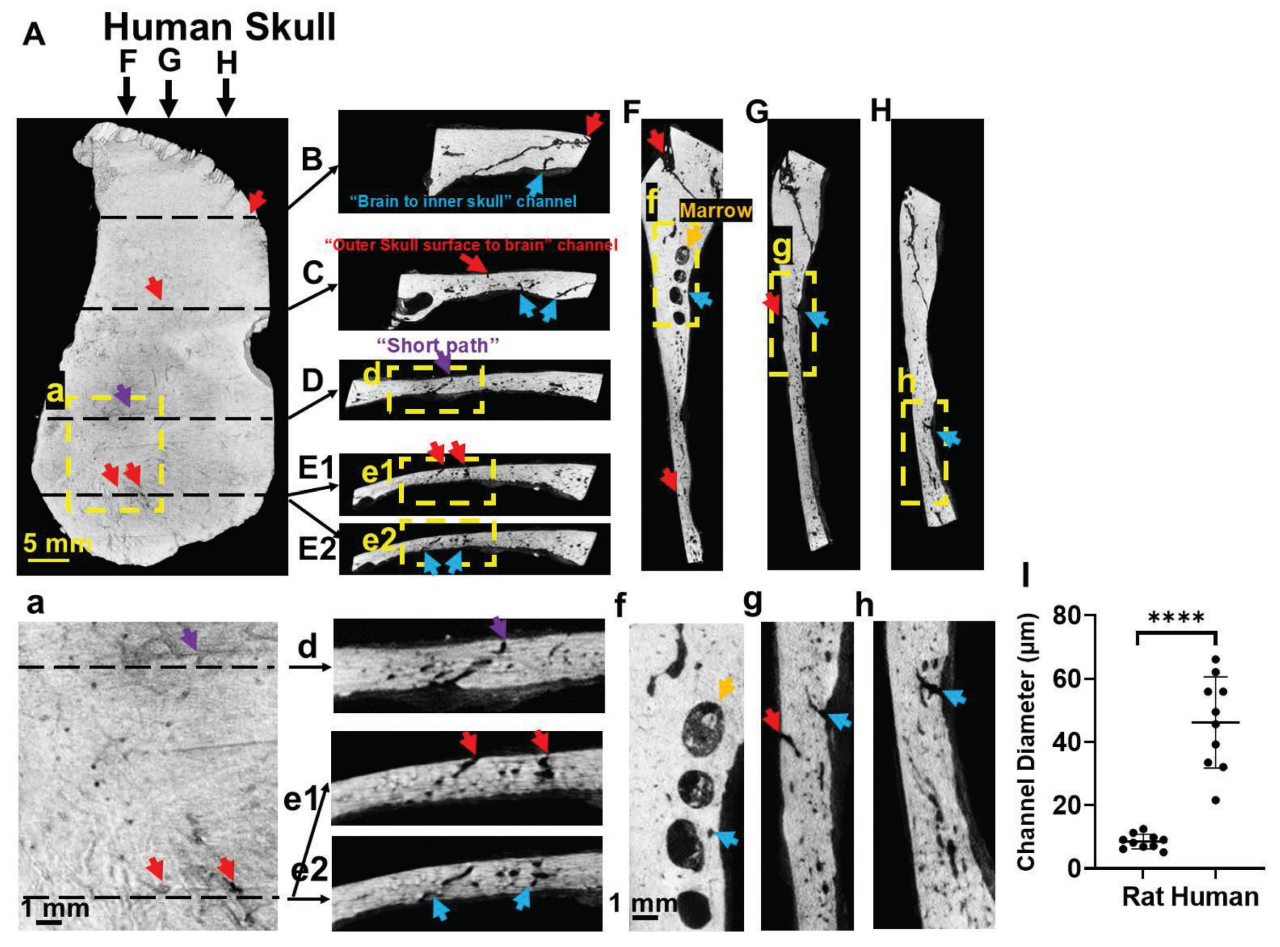
Human skull also shows vessel/marrow pathways connecting outer skull surface and meninges, as reviewed by high-resolution CT. (**A**) Outer skull surface. (**B-E**) Representative coronal views of the skull bone. (**F-H**) Representative sagittal views of the skull bone. (**a**, **d-h**) are enlarged views from the framed locations in (**A**, **D-H**). (**E1**, **E2**) are two contiguous slices to show a surface vessel that goes directly through mid-skull and then a vessel that gets directly into the meninges. (**I**) Quantification of the diameters of skull channels (not including suture line areas) of human skull and rat skull. Red arrows, pores on the outer skull surface (**A**) indicating the “outer skull surface to brain” channels that get to the inner skull. Purple arrows, “short paths” from the skull surface that reach brain meninges (**D**). Blue arrows, “brain to inner skull” channels. Results are expressed as mean ± SD. ****, p < 0.0001.

Human skull also shows many vessel/marrow paths connecting between the outer skull surface and meninges (Figure 3). as detected with microCT images of a piece of human skull. Figure 3E1-E2, e1-e2 are two continuous images to show two surface vessels that pass through mid-brain and connect to a vessel that gets into the meninges. There are also “short paths” in the human skull that directly connect outer skull surface with meninges (Figure 3D,d). The diameters of surface vessels (not including suture line areas) of human skull were 3-5 times larger than that of rat skull, measuring 46.15 ± 14.39 µm in human and 8.15 ± 2.84 µm in rat (Figure 3I).

### Mn^2+^ can be directly delivered through “short paths”

**Figure 4:**
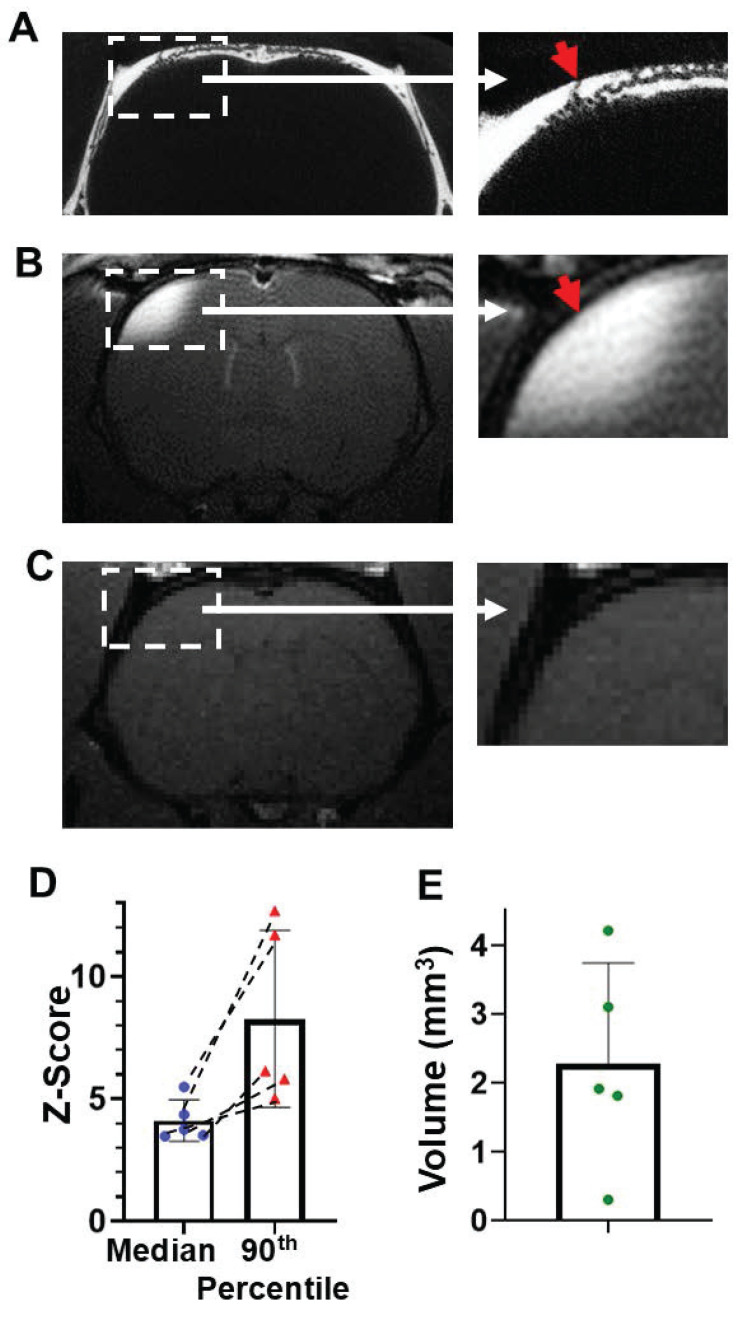
Mn^2+^ can be delivered efficiently through “short paths”. (**A**) Representative coronal view of *in-vivo* microCT image of a short path: a surface vessel that goes directly through mid-skull and then a vessel that gets directly into the meninges. (**B**) T_1_-weighted MRI of this brain area 2-hr post Mn^2+^ application. (**C**) Saline control (n = 4 subjects). (**D**) The average median and 90th percentile of the z-score of the enhanced area was 4.12 ± 0.83 and 8.27 ± 3.62, respectively. Dashed lines connect values from the same subject. (**E**) T_1_ enhanced volume is 2.27 ± 1.47 mm^3^. Data are presented as mean ± SD, n = 5 subjects.

MicroCT images show that there are skull “short paths” that connect a vessel on the outer skull surface, through the mid skull vessel/marrow area, and directly to a vessel that connects to the meninges (Figure 1D,H, 4A). MnCl_2_ solution was pipetted on the top of an opening of a short path of intact skull as identified by prior microCT scan. After 2 hr, Mn^2+^ can be detected in the brain tissue below the “short path” by MEMRI (Figure 4B). Saline control was shown in Figure 4C. The average median and 90^th^ percentile of the z-score of the enhanced area was 4.12 ± 0.83 and 8.27 ± 3.62, respectively (Figure 4 D). T_1_ enhanced volume was quantified to be 2.27 ± 1.47 mm^3^ (Figure 4 E).

### Mn^2+^ can efficiently pass through intact skull above the cerebellum

**Figure 5:**
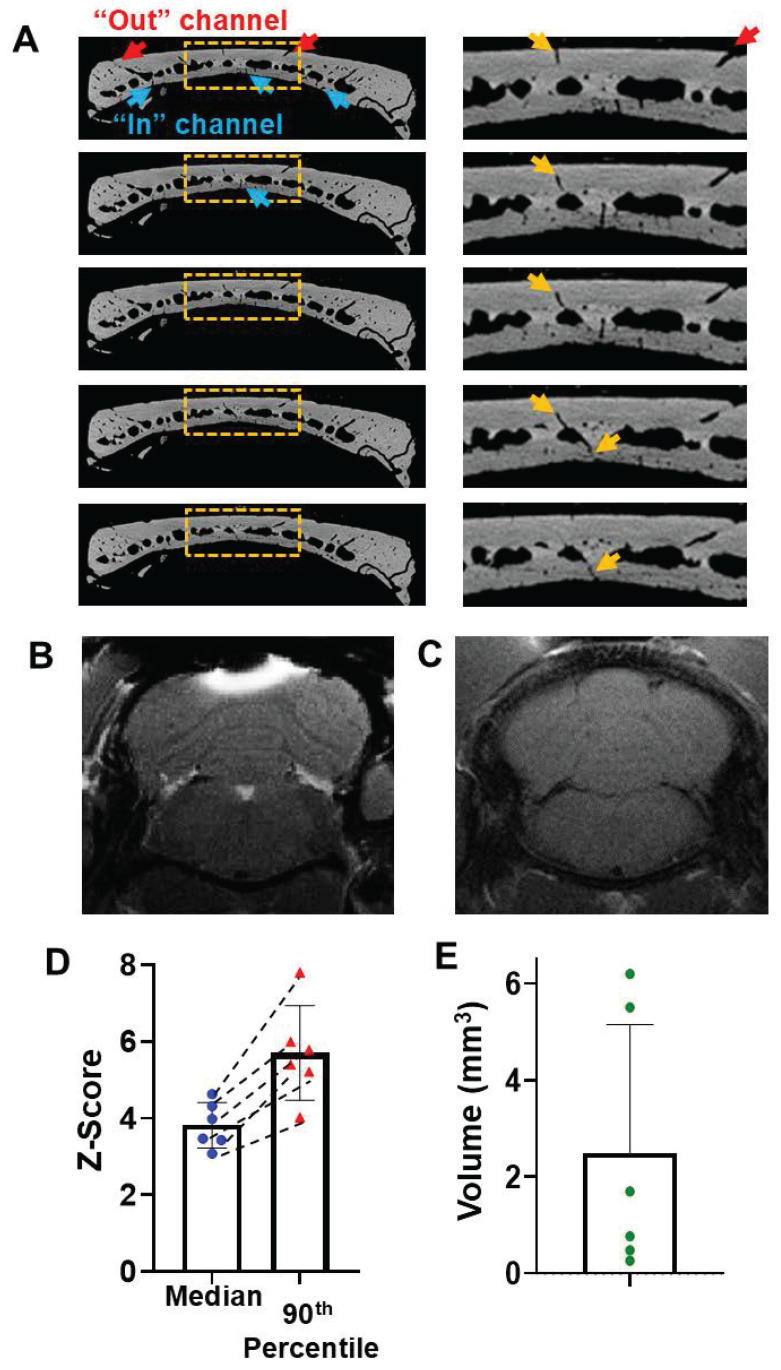
Mn^2+^ can be delivered efficiently through intact skull above the cerebellum. (**A**) Representative coronal views of 5 contiguous *ex-vivo* microCT slices, showing the vessel/marrow pathways connecting from the surface of outer skull to the mid-skull to meninges. (**B**) Representative views of T_1_-weighted MRI of cerebellum 2-hr post Mn^2+^ application. (**C**) Saline control (n = 4 subjects). (**D**) The average median and 90^th^ percentile of z-score of the enhanced area was 3.82 ± 0.59 and 5.70 ± 1.24, respectively. Dashed lines connect values from the same subject. (**E**) T_1_ enhanced volume is 2.48 ± 2.66 mm^3^. Data are presented as mean ± SD, n = 6 subjects.

The efficiency of delivery of Mn^2+^ through the skull when solution was placed above the cerebellum was measured with MEMRI because microCT images showed that this bone areas contains the highest density of surface vessel/marrow paths (Figure 5A). Many surface pores go deeper into the skull and reach the meninges, as shown in the representative views of 5 contiguous slices in Figure 5A. 2-hr post MnCl_2_ solution application on the top of skull, Mn^2+^ can be delivered readily to levels that produce detectable tissue enhancement by MEMRI at cerebellum (Figure 5B). Saline control was shown in Figure 5C. The average median and 90th percentile of the z-score was 3.82 ± 0.59 and 5.70 ± 1.24, respectively (Figure 5D). T_1_ enhanced volume was quantified to be 2.48 ± 2.66 mm^3^ (Figure 5E). H and E staining to show the skull vessel/marrow pathways at the Figure 5B location was shown in Figure S3.

### Minimum thinning of the skull, on the top of a vessel path to the meninges, enables delivery of Mn^2+^ to the brain

**Figure 6:**
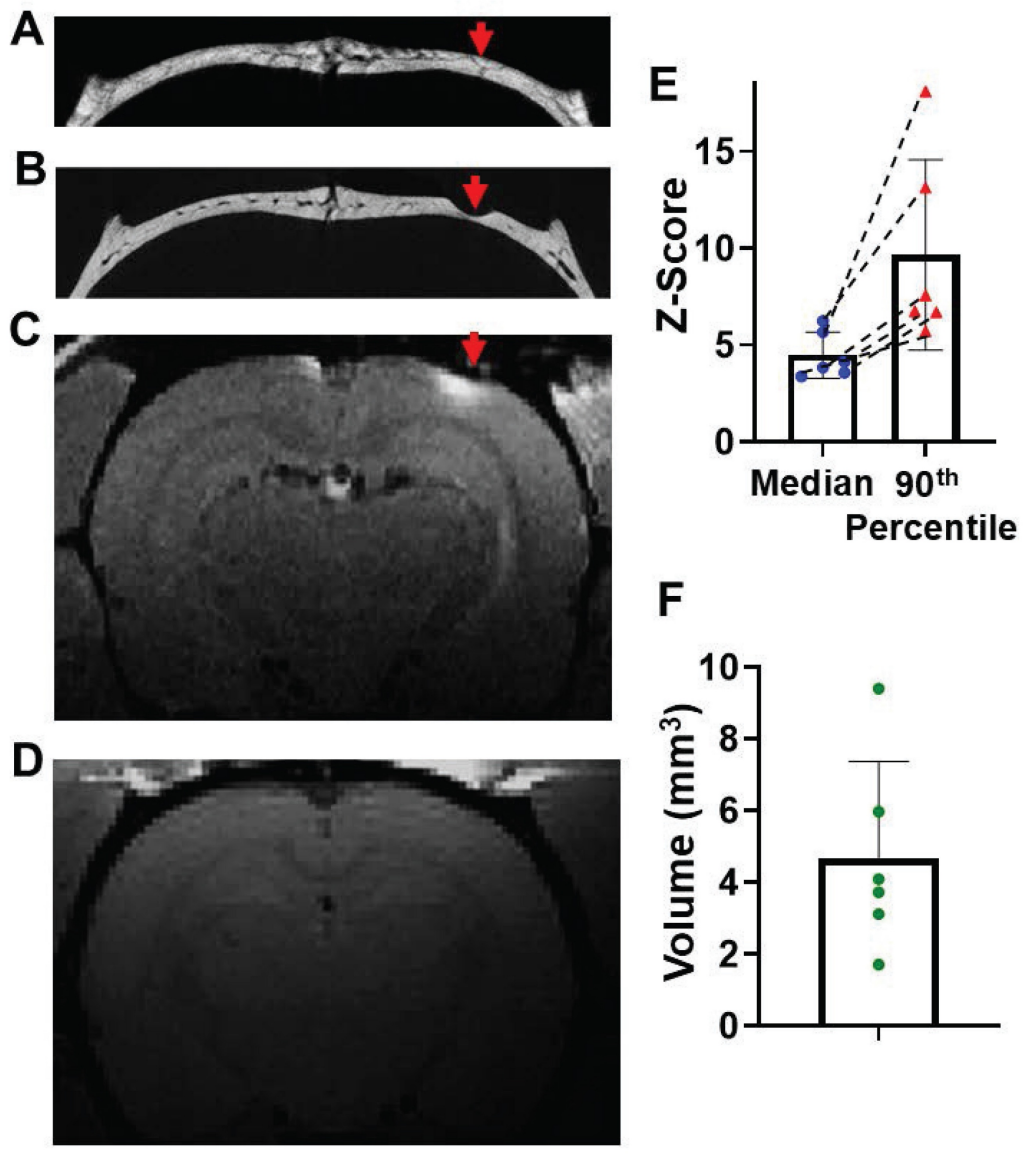
Minimum thinning skull on the top of “brain to skull” vessel/marrow pathways can increase the delivery efficiency. (**A**) Representative *in-vivo* microCT image to identify a “brain to inner skull” vessel/marrow path. (**B**) After thinning the skull at this vessel location, Mn^2+^ was applied. T1-weighted MRI of this brain area 2-hr post Mn^2+^ application. (**C**) Post MRI, rat was subject to another microCT scan to confirm the skull was thinned, but still intact. (**D**) Saline control (n = 6 subjects). (**E**) The average median and 90^th^ percentile of z-score of the enhanced area was 4.8 ± 1.20 and 9.67 ± 4.91, respectively. Dashed lines connect values from the same subject. (**F**) T_1_ enhanced volume is 4.68 ± 2.70 mm^3^. Data are presented as mean ± SD, n = 6 subjects.

Finally, it was tested whether thinning the skull above where a vessel that enters the meninges in areas where there was no path on the upper part of the skull to the skin would enable getting Mn^2+^ into the brain. A vessel path to the meninges was identified from microCT images (Figure 6A) that did not have a vessel path from skull to skin. Skull was thinned to reach this vessel at about halfway through the skull making sure that the skull was still intact where it was thinned (Figure 6B). Through this minimally thinned skull, Mn^2+^ could be delivered efficiently and detected 2-hr post application (Figure 6C). Saline control was shown in Figure 6D. The average median and 90^th^ percentile of z-score was 4.8 ± 1.20 and 9.67 ± 4.91, respectively (Figure 6E). T_1_ enhanced volume was quantified to be 4.68 ± 2.70 mm^3^ (Figure 6F). This thinning skull method was very effective and would be useful especially in areas where direct surface to meninges short paths through the skull are difficult to find.

## Discussion

The brain remains a difficult organ for drug delivery due to the physiological and structural properties of the blood-brain barrier (BBB) and encasement in the skull. New administration routes, such as intranasal and intracranial administrations are gaining interest, but also face challenges 19-21. Transcranial delivery through skull vessel/marrow pathways provides a much less invasive and more local administration route to deliver imaging probes and therapeutics to the brain. Transcranial delivery also bypasses the peripheral circulation and toxicity and increases the bioavailability for brain meninges. As an extension of earlier work 1, 2, this study focused on the brain delivery of Mn^2+^ via specific geometry of vessel/marrow pathways at various skull bone locations, without considering the overall vessel/marrow density in the bone.

There is an intricate geometry of vessel/marrow paths connecting the outer skull surface and meninges, as shown by microCT images (Figures 1 and 3). The meninges consist of epidural space, dura mater, subdural space, arachnoid mater, subarachnoid space [cerebrospinal fluid (CSF)-filled space], and pia mater 22. The vessels in the meninges, especially at the dura mater, are fenestrated and contain less tight junctions compared with those in the brain parenchyma, making them a major point of entry into the meningeal space 23. Acute lymphoblastic leukemia cells were found to migrate into the brain along vessels that pass directly between skull marrow and the subarachnoid space, but were unable to breach the BBB 8. Immune cells were shown to go directly from the skull marrow via vessels into circulation in the brain 4-7. Recently Kipnis et al have demonstrated that injected fluorescence agents can pass along vessels in perivascular spaces that traverse the arachnoid mater into the subarachnoid space 11. Moreover, brain injuries and diseases can disrupt vascular integrity in the meninges, resulting in the leakage of materials, such as gadolinium-based MRI contrast agent, into the subarachnoid space. An MRI technique, known as hyperintense acute reperfusion marker (HARM), is a delayed enhancement of the subarachnoid space observed on post-contrast fluid-attenuated inversion recovery (FLAIR) images and is associated with permeability changes to the BBB caused by Stroke or TBI 24, 25. MRI data also showed leak of contrast into the subarachnoid space in older, healthy people 11.

Here we show that these vessel/marrow pathways provide a pathway for MRI contrast agent into the meninges and then to the brain from outside the skull. After a simple application of MnCl_2_ on the outer skull surface, Mn^2+^ can be detected in the brain parenchyma by MEMRI, most efficiently at the high vessel/marrow density location, through “short paths”, or cerebellum (Figures 4 and 5). Many diseases are associated with cerebellum, such as medulloblastoma 26, 27 and cerebellar syndrome 28. Transcranial delivery might be very useful for the delivery of imaging agents for disease mechanistic studies and therapeutics for managing the diseases associated with cerebellum, since the skull areas above the cerebellum showed the highest density of vessel/marrow pathways (Figure 1).

Direct “short paths” (Figure 1D,H) are also very efficient sites for transcranial delivery. MicroCT scan is required to identify these “short paths” for each rat, since the distributions of skull vessel/marrow pathways are unique for individual rat. “Short paths” might be seen on one microCT slice or continuous through several slices. In this study, MnCl_2_ solution was dripped on the surface pore of a “short path”. In future study, an injection catheter equipped with a pump and with ideal flexibility, going through this “short path”, shall improve the delivery efficiency significantly. The larger size of skull vessel/marrow pathways in humans (Figure 3) is expected to make this delivery pathway even more efficient and useful for human patients.

Mn^2+^ is an essential element and nutrient for the normal functioning of an organism 29, 30. Mn^2+^shows paramagnetic properties and can enter excitable cells through voltage-gated calcium channels 31, 32 as well as through iron and zinc transporters. Recently dominant Mn^2+^ transporters have been described and shown to be expressed throughout brain and brain vessels 33. Due to this rich biology of Mn^2+^, MEMRI has been used broadly in preclinical studies to image tissue structure, to study activity in brain, heart and pancreas and to perform neural tracing in the brain 34-39. The application of MEMRI in preclinical animal models of CNS diseases has provided important information for the study of disease mechanisms 40, 41. Thus, the full range of uses of MEMRI should be available with transcranial delivery. Indeed, an earlier study demonstrated that once in the brain parenchyma, Mn^2+^ introduced through transcranial delivery can be used for neuronal tract tracing 2.

It is not clear how Mn^2+^ got from the meninges into the brain parenchyma as clearly detected with MEMRI. It could be that Mn^2+^ (500 mM) are of high osmolarity which enabled Mn^2+^ to cross through the meninges into the brain. Alternatively, it could be that Mn^2+^ was able to cross through the dura mater and arachnoid mater through transport pathways into the subarachnoid space. It has been proposed that Mn^2+^ leaking through fenestrated surface vessels in the meninges can cross into the brain and this would imply transport through the dura and arachnoid cell layer 2. Choroid plexus is known to transport Mn^2+^ from brain into CSF 42. Finally, it could be that Mn^2+^ can follow vessels that have recently been shown to cross from the dura mater into the subarachnoid space and possibly into the brain 11.

## Conclusion

Mn^2+^ can be delivered to the brain by simple application on the rat skull especially if care is taken to use the vessel/marrow structure in the brain to enhance deliver. Short vessel marrow pathways that go directly through the skull can be used to efficiently deliver Mn^2+^ but require CT to identify prior to application. We also demonstrated that another area with dense vessel marrow, the cerebellum, direct application to the skull is efficient for delivery in support of earlier studies. Finally with minimal skull thinning numerous vessels through the brain can be identified and used for delivery. Development of a thin flexible catheter capable of passing through the skull marrow/vessel paths would enable direct injection through the skull in almost any area without needing to thin the skull. Thus, there is much potential in using transcranial delivery of small molecules for therapeutic, basic biology, and delivery of imaging agents.

## Supplementary Material

Supplementary figures, table, and video legend.

Supplementary video.

## Abbreviations

ANT: Advanced Normalization Tools
BBB: the blood-brain barrier
CSF: cerebrospinal fluid
FLAIR: fluid-attenuated inversion recovery
FOV: field of view
HARM: hyperintense acute reperfusion marker
MEMRI: manganese-enhanced MRI
microCT: micro computed tomography
Mn^2+^: manganese ion
