# Supplementary material for: Direct delivery of MRI contrast through skull vessel/marrow pathways into the brain guided by microCT
Source: Theranostics. 2025 Jun 9;15(14):6615–27. doi: 10.7150/thno.117250 (PMC12203665; doi:10.7150/thno.117250)
Supplement: Supplementary file 1 — Supplementary figures, table, and video legend. [file thnov15p6615s1.pdf]

Supplementary Information

Direct delivery of MRI contrast through skull vessel/marrow pathways into the brain guided by microCT

Li Liu[1,*], Martin J. MacKinnon[1], Tatjana Atanasijevic[1], Stephen Dodd[1], Nadia Bouraoud[1], Danielle Donahue[2], Harikrishna Rallapalli[1], Alan P. Koretsky[1,*]

[1]Section on Plasticity and Imaging of the Nervous System, Laboratory of Functional and Molecular Imaging, National Institute of Neurological Disorders and Stroke, National Institutes of Health, Bethesda, MD 20892, USA.

[2]Mouse Imaging Facility, National Institute of Neurological Disorders and Stroke, National Institutes of Health, Bethesda, MD 20892, USA.

*Corresponding to: li.liu3@nih.gov; koretskya@ninds.nih.gov.

**Figure**	**Experiment**	**Animal Number**
1	Quantification of volume ratio of channel (total of skull surface to brain and brain to skull) to bone and marrow to bone	6
2	H and E staining of rat skull bone	6
4	Mn^{2+} can be delivered efficiently through "short paths"	
	Mn^{2+} delivery	5
	Saline control	4
5	Mn^{2+} can be delivered efficiently through intact skull above the cerebellum	
	Mn^{2+} delivery	6
	Saline control	4
6	Minimum thinning skull can increase the delivery efficiency	
	Mn^{2+} delivery	6
	Saline control	6
Supplementary Video 1	The reconstructed 3D microCT image to show the intricate geometry of vessel/marrow paths.	1
S1	MicroCT of bottom skull of a rat, especially under the olfactory bulb and forebrain, showed a large volume of marrow	3
S2	H and E staining of rat skull bone and femur bone marrow	1
**Total**		**48**

Table S1. Animal number used in each experiment.

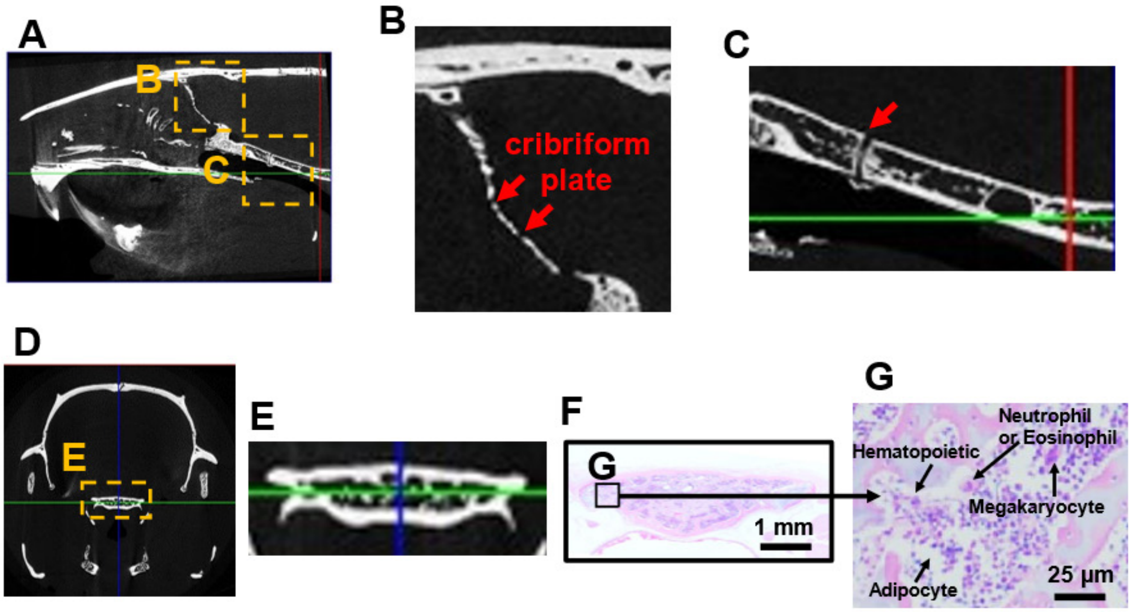

Figure S1. Bottom skull of a rat, especially under the olfactory bulb and forebrain, showed a large volume of marrow. (**A-C**) microCT images of sagittal view of bottom skull. (**D, E**) microCT images of coronal view of bottom skull. (**F, G**) H & E staining of the marrow in the bottom skull.

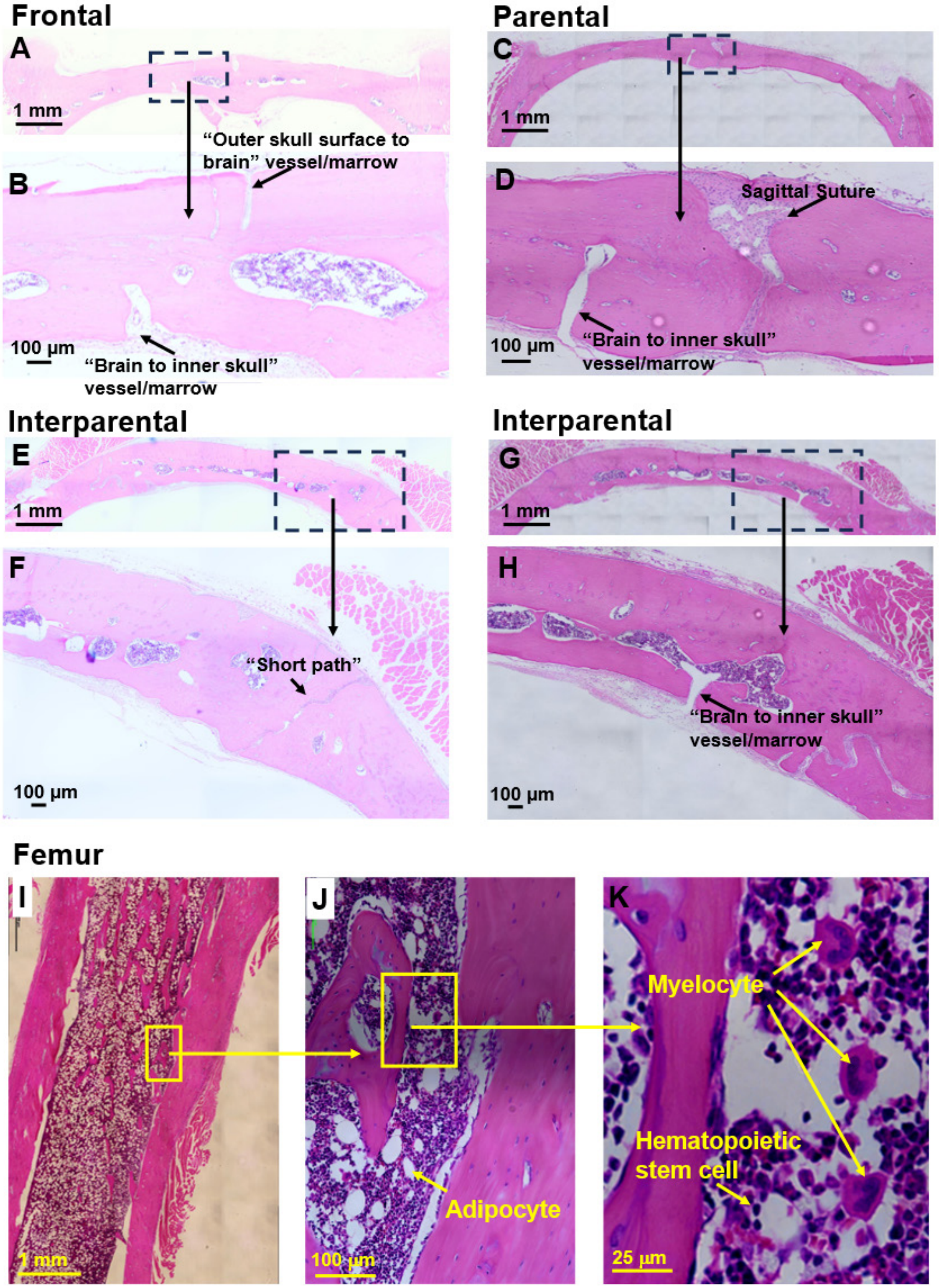

Figure S2. H and E staining of rat skull bone and femur bone marrow. (**A,B**) Frontal bone. (**C,D**) Parietal bone. (**E-H**) Interparietal bone. (**I-K**) Femur bone.

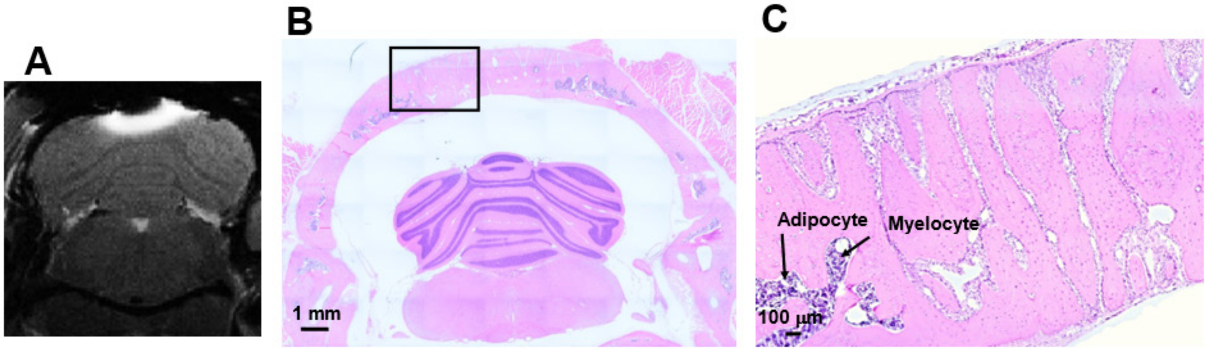

Figure S3. Efficient delivery of Mn^{2+} through intact skull above the cerebellum by using the skull/marrow pathways. (**A**) Representative views of T_1-weighted MRI of cerebellum 2-hr post Mn^{2+} application. (**B, C**) H and E staining of the skull-brain tissue at the cerebellum to show the skull vessel/marrow pathways.

Data Availability Statement

Figure-Data-List through Figshare
Link: https://figshare.com/s/0c348af11fb86ab9e9b1

Figure 1.Zip High-resolution microCT of a rat top skull showing an intricate geometry of vessel/marrow paths connecting the outer skull surface and meninges.
Link: https://figshare.com/s/5fb97feeef60a24f36a0
Figure-1-(A-K) Coronal views of high-resolution microCT of a rat top skull and the reconstructed 3D image to show the intricate geometry of vessel/marrow paths.
Figure-1-(L-O) Quantification of volume ratio of channel (total of skull surface to brain and brain to skull) to bone and marrow to bone.

Figure 3.Zip Human skull also shows vessel/marrow pathways connecting outer skull surface and meninges, as reviewed by high-resolution CT.
Link: https://figshare.com/s/c8a42bf566d25bd3e719
Figure-3-(A-H) Coronal views of high-resolution CT of a human skull shows vessel/marrow pathways connecting outer skull surface and meninges.
Figure-3-I Quantification of the diameters of skull channels (not including suture line areas) of human skull and rat skull.

Figure 4.Zip Mn^{2+} can be delivered efficiently through "short paths".
Link: https://figshare.com/s/ed5759286245f9499ace
Figure-4-B Representative T_1-weighted MRI to show that Mn^{2+} can be delivered efficiently through "short paths".
Figure-4-D Data for the quantification of the average median and 90th percentile of the contrast-to-noise ratio (CNR) of the enhanced area.
Figure-4-E Data for the quantification of T1 enhanced volume.

Figure 5.Zip Mn^{2+} can be delivered efficiently through intact skull above the cerebellum.
Link: https://figshare.com/s/8e8fa297fd8e93d59a9f
Figure-5-B Representative T_1-weighted MRI to show that Mn^{2+} can be delivered efficiently through intact skull above the cerebellum.
Figure-5-D Data for the quantification of the average median and 90th percentile of the contrast-to-noise ratio (CNR) of the enhanced area.
Figure-5-E Data for the quantification of T1 enhanced volume.

Figure 6.Zip Minimum thinning skull on the top of "brain to skull" vessel/marrow pathways can increase the delivery efficiency.
Link: https://figshare.com/s/b7076ed02275184a0f4c
Figure-6-B Representative T_1-weighted MRI to show that minimum thinning skull on the top of "brain to skull" vessel/marrow pathways can increase the delivery efficiency.
Figure-6-E Data for the quantification of the average median and 90th percentile of the contrast-to-noise ratio (CNR) of the enhanced area.

Figure-6-F Data for the quantification of T1 enhanced volume.

Supplementary Video 1. The reconstructed 3D microCT image to show the intricate geometry of vessel/marrow paths.
Link: https://figshare.com/s/e506d8567974069184a9
